# Supplementary material for: Modeling Key Characteristics of Rigid Polyisocyanurate Foams to Improve Sandwich Panel Production Process
Source: Materials (Basel). 2025 Feb 17;18(4):881. doi: 10.3390/ma18040881 (PMC11857549; doi:10.3390/ma18040881)
Supplement: Supplementary file 1 [file materials-18-00881-s001.zip › materials-3411680-supplementary.pdf]

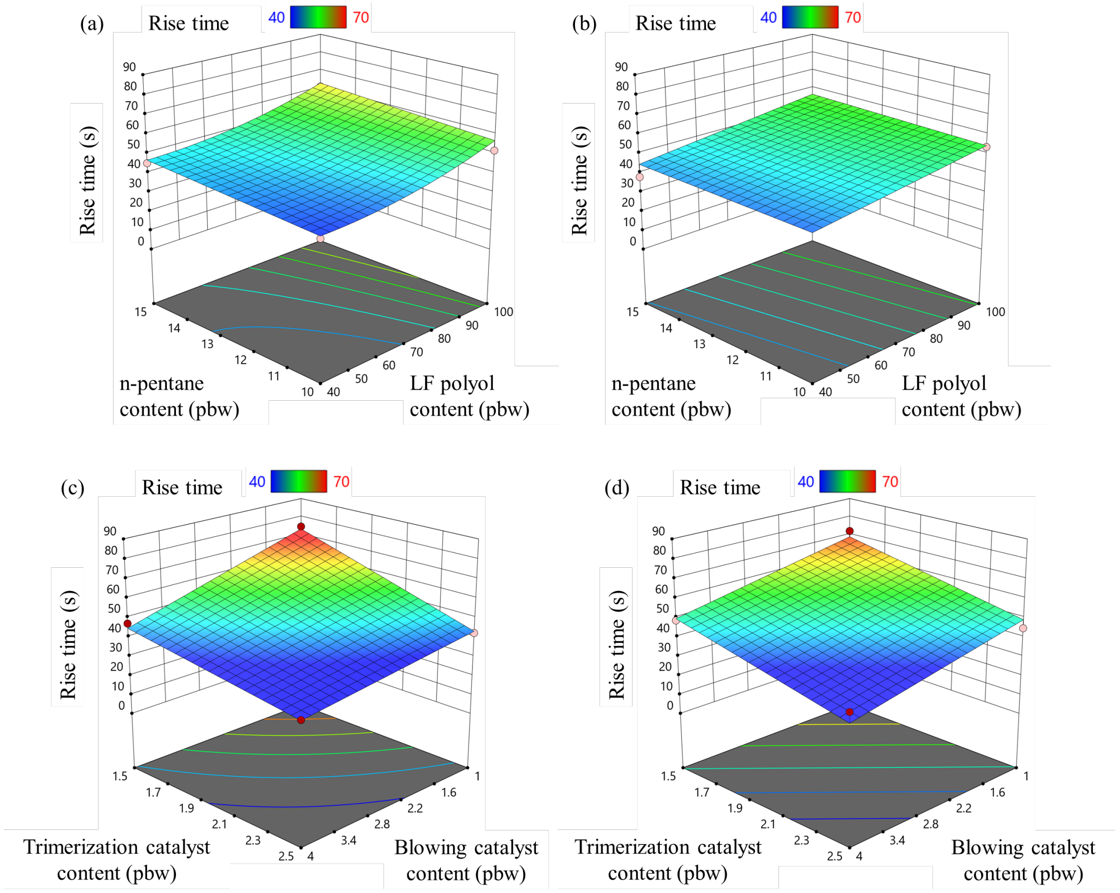

Figure S1. LF polyol and n-pentane influence on foaming rise time for rigid PIR foam with isocyanate index a) 335 and b) 400; trimerization and blowing catalyst influence on foaming rise time for rigid PIR foam with isocyanate index c) 335 and d) 400

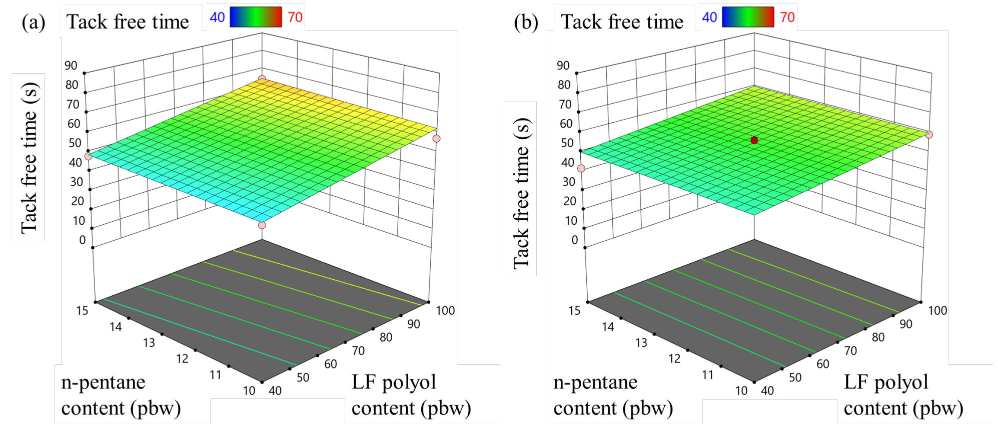

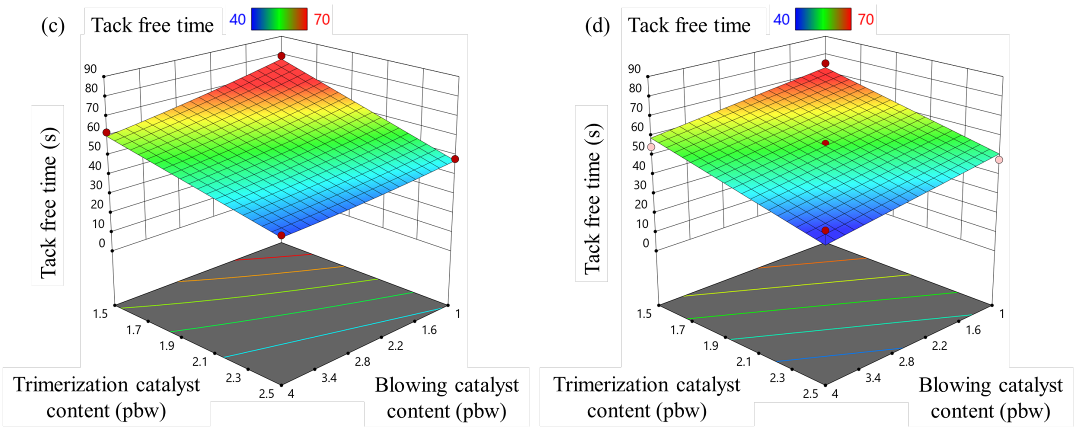

Figure S2. LF polyol and n-pentane influence on foaming tack free time for rigid PIR foam with isocyanate index a) 335 and b) 400; trimerization and blowing catalyst influence on foaming tack free time for rigid PIR foam with isocyanate index c) 335 and d) 400

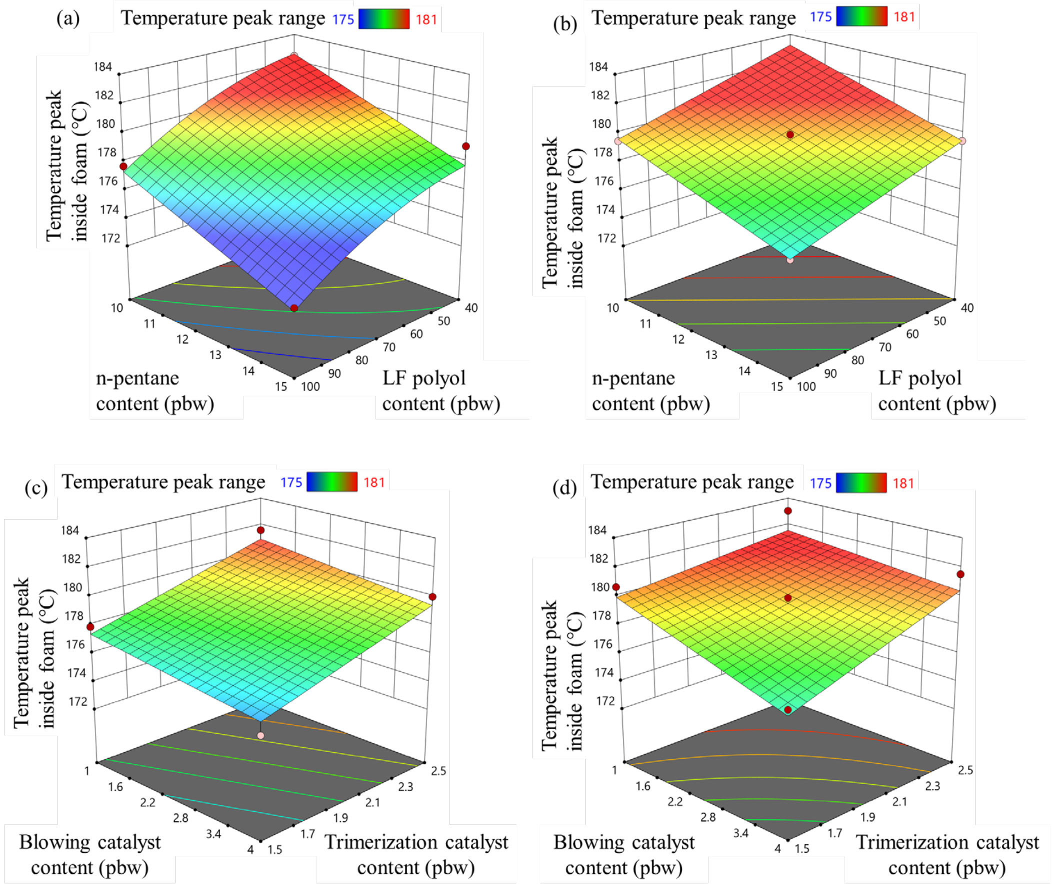

Figure S3. LF polyol and n-pentane influence on temperature inside foams block for rigid PIR foam with isocyanate index a) 335 and b) 400; trimerization and blowing catalyst influence on temperature inside foams block for rigid PIR foam with isocyanate index c) 335 and d) 400

Table S1. The changed factors of the RSM and the measured responses, start time, gel time, rise time, tack free time, temperature peak during foaming, apparent density, thermal conductivity, compression strength and compression modulus for rigid PIR foams with isocyanate index 335.

Factor 1	Factor 2	Factor 3	Factor 4	Response 1	Response 2	Response 3	Response 4	Response 5	Response 7	Response 8	Response 9	Response 10
A:LF polyol content	B:Blowing catalyst	C:Trimerization catalyst	D:n-pentane	Start time	Gel time	Rise time	Tack free time	Temperature peak	Apparent density	Thermal conductivity	Compression strength	Compression modulus
pbw	pbw	pbw	pbw	s	s	s	s	C	kg/m^3	mW/(m·K)	MPa	MPa
100	1	2	12.5	19.6	59.3	72	67.2	175.6	38.2	23.4	0.252	5.05
100	4	2	12.5	11.5	34.9	49	59.5	174.4	36.7	23.11	0.254	5.87
100	2.5	1.5	12.5	15.9	55.4	75.8	81.4	173	38.3	23.55	0.257	5.57
100	2.5	2	15	13.4	48.4	60.9	64.3	172.7	34.5	23.53	0.224	4.77
100	2.5	2.5	12.5	13.6	37.3	49.9	54.1	175.9	37.8	23.42	0.262	5.54
100	2.5	2	10	13	38.3	51.9	57.3	177.7	43.3	22.72	0.312	6.72
70	2.5	2	12.5	11.6	30.8	44.3	52	178	41	23.28	0.296	6.27
70	1	2	15	17.8	40.1	62.9	61.6	176.5	36.9	23.53	0.246	5.3
70	2.5	2.5	10	11.5	25.2	38.3	44.4	182.7	39.7	22.89	0.29	6.94
70	4	2.5	12.5	9.3	19.9	32	42.3	180	39.8	~~24~~	0.266	5.3
70	1	2	10	17.8	41.3	53.7	61.2	182	46.3	22.87	0.337	7.56
70	4	2	10	9.8	23.8	37.4	49.9	180.5	45.5	22.92	0.347	7.01
70	1	1.5	12.5	21.3	59.2	74.5	79.2	177.9	42	23.44	0.301	6
70	1	2.5	12.5	14.5	32.9	42.5	48.5	181.6	40.1	23.33	0.273	5.73
70	4	2	15	9.7	23	37.5	49	177.1	36.6	23.22	0.256	5.35
70	2.5	2.5	15	10.6	28.3	41.1	44.4	176.5	35.9	~~23.1~~	0.251	5.38
70	2.5	1.5	15	13.6	48.4	62.6	70.8	173.2	36.6	23.47	0.295	6.74
70	2.5	2	12.5	12.1	30.5	48.2	54.5	177.9	41.6	23.04	0.255	~~4.89~~
70	4	1.5	12.5	10.3	31.7	47.4	62.1	175	41.2	23.38	0.312	6.82
70	2.5	1.5	10	12.8	34.2	54.1	66.3	178.7	49.1	23.29	0.371	7.48

40	2.5	2.5	12.5	13.9	22.7	34	38.6	181.1	48.2	23.53	0.353	7.28
40	2.5	1.5	12.5	13.5	34.2	52.5	58.3	178.8	47.7	22.84	0.392	8.38
40	4	2	12.5	10.4	20.3	37.4	41.3	178.5	45.4	22.99	0.362	8.04
40	2.5	2	15	12.3	32.1	45.4	48.2	179.1	39.9	23.26	0.308	6.62
40	1	2	12.5	19.5	43.4	55	57.6	181	45.0	23.15	0.34	7.04
40	2.5	2	10	11.5	25.5	39.9	45.5	182.4	50.3	22.98	0.412	8.76

Table S2. The changed factors of the RSM and the measured responses, start time, gel time, rise time, tack free time, temperature peak during foaming, apparent density, thermal conductivity, compression strength and compression modulus for rigid PIR foams with isocyanate index 400.

Factor 1	Factor 2	Factor 3	Factor 4	Response 1	Response 2	Response 3	Response 4	Response 5	Response 7	Response 8	Response 9	Response 10
A:LF polyol content	B:Blowing catalyst	C:Trimerization catalyst	D:n-pentane	Start time	Gel time	Rise time	Tack free time	Temperature peak	Apparent density	Thermal conductivity	Compression strength	Compression modulus
pbw	pbw	pbw	pbw	s	s	s	s	C	kg/m^3	mW/(m·K)	MPa	MPa
100	1	2	12.5	17.8	60.6	71.1	72.0	178.8	43.2	23.93	0.282	6.15
100	4	2	12.5	8.9	32.9	46.4	56.8	177.9	42.9	23.81	0.274	6.58
100	2.5	1.5	12.5	12.9	53.3	66.9	74.8	175.9	44.3	23.76	0.288	6.23
100	2.5	2	15	9.0	35.7	45.6	46.8	175.8	39.7	23.60	0.262	6.17
100	2.5	2.5	12.5	9.0	36.3	46.9	49.6	178.5	42.6	23.42	0.287	6.60
100	2.5	2	10	9.3	41.6	53.8	59.3	179.5	49.5	23.73	0.355	8.28
70	2.5	2	12.5	11.0	32.1	46.6	56.4	179.1	46.4	23.72	0.339	7.83
70	1	2	15	18.8	53.4	66.0	66.3	179.2	42.0	24.06	0.278	6.29
70	2.5	2.5	10	11.8	30.0	42.2	45.8	180.8	47.0	23.51	0.330	7.43
70	4	2.5	12.5	10.3	24.9	35.7	44.6	181.5	52.4	23.49	0.387	9.69
70	1	2	10	17.2	43.2	54.0	61.5	183.6	53.3	23.51	0.387	8.40
70	4	2	10	10.0	25.2	37.2	45.0	180.1	53.8	23.33	0.434	8.90
70	1	1.5	12.5	20.4	58.8	72.0	75.0	180.7	57.4	24.08	0.419	8.85
70	1	2.5	12.5	13.9	33.4	45.0	48.1	183.1	45.8	23.55	0.319	7.05
70	4	2	15	9.8	29.9	42.6	52.6	176.3	42.8	23.63	0.305	6.53

70	2.5	2.5	15	11.9	30.6	42.1	44.5	178.4	41.1	23.41	0.282	6.37
70	2.5	1.5	15	14.2	51.7	65.5	73.6	174.8	42.4	24.00	0.299	6.84
70	2.5	2	12.5	11.4	33.2	44.5	45.8	179.9	46.3	23.49	0.331	7.53
70	4	1.5	12.5	10.8	35.5	49.0	54.7	176.6	48.8	23.45	0.365	8.19
70	2.5	1.5	10	12.3	42.0	57.4	62.3	179.3	55.7	23.99	0.440	9.57
40	2.5	2.5	12.5	10.8	25.9	35.7	41.8	182.3	49.1	23.52	0.368	7.20
40	2.5	1.5	12.5	13.4	38.6	53.4	63.1	179.9	52.2	23.65	0.391	8.43
40	4	2	12.5	10.6	25.2	37.0	46.0	180.8	50.8	23.36	0.394	9.53
40	2.5	2	15	11.5	27.4	38.2	42.0	179.5	53.9	24.77	0.394	8.98
